# Layer Bond Strength in 3D-Printed Concrete: The Role of Interlayer Surface Area and Printing Delay Time

**DOI:** 10.3390/ma19061168

**Published:** 2026-03-17

**Authors:** Nikol Žižková, Josef Válek, Arnošt Vespalec, Jindřich Melichar, Sławomir Czarnecki, Adrian Chajec

**Affiliations:** 1 Faculty of Civil Engineering, Institute of Technology of Building Materials and Components, Brno University of Technology, Veveří 331/95, 602 00 Brno, Czech Republic; nikol.zizkova@vut.cz (N.Ž.); jindrich.melichar@vut.cz (J.M.); 2 Faculty of Civil Engineering, Brno University of Technology, Veveří 331/95, 602 00 Brno, Czech Republic; arnost.vespalec@vut.cz; 3 Faculty of Civil Engineering, Wroclaw University of Science and Technology, Wybrzeże Wyspiańskiego 27, 50-372 Wrocław, Poland; slawomir.czarnecki@pwr.edu.pl (S.C.); adrian.chajec@pwr.edu.pl (A.C.)

**Keywords:** 3DCP, additive manufacturing, concrete printing, interlayer surface area, bond strength, homogenous mechanical properties

## Abstract

**Highlights:**

**What are the main findings?**

- Interlayer bond strength increases linearly with surface area up to a threshold.
- A 20% increase in surface area yielded up to 70% higher bond strength.
- The relative strength gain remained consistent across delay times.
- Bond strength shows a logarithmic dependence on delay time.

**What are the implications of the main findings?**

- Surface modification is effective only within a defined range.
- The threshold depends on material properties and teeth geometry.
- Retractable teeth may reduce interlayer-induced heterogeneity.
- The method can improve mechanical uniformity in printed elements.

**Abstract:**

Three-dimensional (3D) printing, also known as additive manufacturing of cementitious materials, appears to be a promising way to build in a way that is more time-efficient, cost-effective and, under certain conditions, environmentally friendly. This technology continues to exhibit significant inhomogeneity, which is frequently caused by the interlayer area. The presented research aims to clarify the influence of the interlayer surface area and delay time on the bond strength. This study involved reference cast and printed samples with different delay times and cast samples with different interlayer surface areas. Different interlayer surface areas were accomplished through the utilisation of a teeth shaper before casting the second layer. Research has shown that the interlayer surface area has a significant impact on layer bond strength; up to a 70% increase in bond strength can be achieved while increasing the area by 20%. The results show that the increase in strength due to a larger surface area remained constant in terms of percentage, across delay times, with a linear dependency on a specific range of conditions. After the threshold of the surface area increased, the bond strength could be compromised and lowered. This threshold is above a 120% increase in surface area for the used teeth geometry and material. The proposed technology of ejecting teeth to alter the interlayer surface area has the potential to reduce the heterogeneity of mechanical properties in 3D-printed objects, caused by the different delay time between layers, because of the print strategy or material shortage.

## 1. Introduction

So-called additive manufacturing (AM) is experiencing a major boom across industries and in utilised materials. This alternative route generally allows for more ecological and economical manufacturing. Since cement-based materials (concrete, mortar) are among the most widely used materials, there are efforts to use additive manufacturing methods with those materials as well. To date, three main methods of manufacturing cement-based materials have been distinguished: (i) 3D concrete printing (3DCP), (ii) 3D contour crafting (3DCC), and (iii) D-shape [1]. The 3D printing (a synonym for additive manufacturing, not a specific manufacturing method) of cement-based materials theoretically produces less waste and lower material consumption. The decrease in construction material consumption can reduce water content and the CO_2_ emissions caused by the hydration of cement. It is believed that the advancement of 3D printing could revolutionise the construction industry of the twenty-first century [2] or help in the colonisation of space [3].

These possibilities are enabled by the basic principle of 3D printing (AM). A suitable printable mixture [2] is prepared and delivered to the nozzle using a mixer and a pump. The nozzle of the printing machine can be positioned using different types of mechanisms (e.g., a robotic arm or a gantry printer). When evaluating the suitability of a particular printing machine, several factors must be considered [4]. The well-known process of cement hydration and hardening of the mixture begins after the initial mixing of the dry input materials with water (in the mixer prior to transport to the nozzle). The chemical principles governing cement hydration are the same in traditional construction and in 3D printing; however, differences in the build strategy (layer-by-layer-wise material addition) require consideration of several additional parameters.

To fully exploit the potential of 3D-printing technology in cementitious materials, further research and development are required in several identified areas. Significant advancement must be made in the field of mechanical equipment, including pumps, mixing machines, nozzles, and other similar devices. Each of these components contributes to the production chain, which has a significant impact on the mechanical properties of the manufactured component [5,6]. Another area that requires improvement is the prediction of printing outcomes, particularly through computer simulations. The ability to predict the material behaviour and mechanical properties of the printed structure based on material properties, environmental conditions (temperature, humidity and air flow), and printing such as geometry and printing strategy is a key factor for the successful development of 3DCP technology. To predict this behaviour, further research and development are required in the fields of material properties [2,7,8,9], testing methods [10], and numerical simulations [11]. A mixture suitable for 3DCP [2,8], characterized by reduced CO_2_ emissions [12] and enhanced mechanical properties, has previously been introduced. However, these benefits may be diminished by the influence of the printing process and the limited ability to predict the final printing outcome. Furthermore, the previously mentioned influencing factors are concentrated within a small volume of material located between two layers. This interlayer area region is highly sensitive to a wide range of parameters (printing parameters, material properties, and environmental conditions) and has a substantial impact on the overall mechanical performance of the printed structure.

Open time and delay time are among the main factors influencing the mechanical properties of the interlayer bond [13]. Open time refers to the period during which a sufficient bond between layers can develop while hydration of the material progresses. Delay time represents the time interval between printing a subsequent layer onto the previous one. While open time is a material property, delay time is a printing parameter. These parameters must be properly matched.

Bonding of material is significantly affected by surface roughness [14,15,16,17], porosity [13] and the curing method for concrete.

The microstructure of the interlayer area, including pore distribution and geometry, is influenced by mixture composition, including the incorporation of recycled materials and fibres [18]. The size and quantity of pores are also influenced by print parameters, including single-layer print height, feed rate, and delay time but also mixture composition [13,18,19], and can vary across samples [20]. Porosity appears to be an important part of bond formation under uniaxial tension, due to the layers anchoring in the pores [17], which also affects the failure pattern. From this, it can be concluded that when the surface layer is treated differently, e.g., while creating a more complex geometry, there will be differences in the microstructure, and, thus, the mechanism of damage (and overall mechanical performance) will also differ.

Methods for modifying layers after printing include modifying evaporation and bleeding of water from the mixture [21,22,23,24,25]. Water bleeding, which adversely affects the interlayer bond, was observed also in a study conducted by Marchment [26]. The study identified the influence of the interlayer surface area on material bonding, through mechanical anchorage. Similarly, Panda [27] suggested improving interlayer bonding by increasing the surface area at the interface. While certain authors investigated the bonding of concrete interfaces with varying roughness (contact surface area), the behaviour under conditions of 3D printing has not been sufficiently researched yet (Zhao [28] specifically studied concrete repair systems and discovered linear dependency between splitting tensile strength and the interlayer roughness). The modification of interlayer geometry is also a major topic in the current literature, and several different approaches can be found [6,29,30].

A contrast to the previous paragraphs can be observed. Increasing the surface area, as suggested, can without doubt lead to an increase in the bond strength. Simultaneously, this will lead to an increase in the evaporation rate and porosity. Increased evaporation and, thus, drying of the mixture and increased porosity will compromise the mechanical properties of the hardened material. It is unclear which of these phenomena will have a greater impact when altering the contact surface area of layers on a larger scale than just the roughness.

The novelty of the presented study lies in investigating the combined influence of interlayer geometry and delay time on the mechanical properties of the material in the cured state. Understanding the influence of these two key variables of printing parameters is a crucial and sensitive aspect for additive manufacturing with cementitious materials.

## 2. Materials and Methods

This research uses a recently developed mixture, as explained in Section 2.1. When investigating the impact of various printing and design factors on cementitious materials, there are two potential approaches that can be employed: (a) Printing material, as in the application, has the advantage in realistic AM process, but changing only one parameter and trying to deduce conclusions can be more complex (used by [6,27,31]), as the results can be affected, e.g., by rheology in the nozzle [2]. (b) Casting samples (with the required properties) is a much easier approach, with better controllable conditions (used by [13]). Casting samples was found to be the most suitable method for accurately investigating the influence of surface area on layer strength. However, it is necessary to verify sufficient similarity between printed and cast samples in terms of bond strength. The authors, therefore, also investigated the bond strength and microstructure (details in Section 2.5) in the interlayers of cast and printed samples (see Section 3.1) with basic geometry (geometry A, see Section 2.2).

### 2.1. Mixture

Portland cement CEM I was excluded from the selection of binders considered, due to its high energy demand and associated ecological and economic burden. Owing to legislation related to the European Green Deal from 2019, which, among other measures, plans to reduce greenhouse gas emissions by 2030, it is necessary to decrease the amount of clinker used. Therefore, mixed Portland cement was used as a silicate binder. According to EN 197-1 [32], this type of cement consists of Portland clinker and other components, especially latent hydraulic components. Commonly used additives are fly ash, slag, or limestone.

From the range of binders considered, CEMII/B-M(S-LL) 32.5 R from Českomoravský cement, a.s. was selected as the most suitable cement from the Mokrá production plant (Czech Republic). Its mineral composition was identified via X-ray powder diffraction (XRD, Table 1). The main minerals identified by XRD analysis include alite (C_3_S), belite (C_2_S), ferrite (C_4_AF), aluminate (C_3_A), calcite (CaCO_3_) and gypsum (CaSO_4_∙2H_2_O).

**Figure 1:**
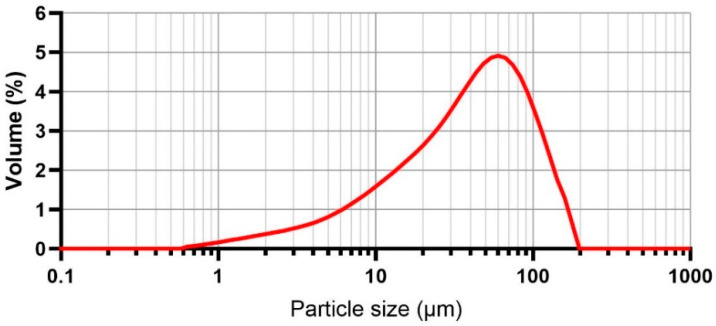
Particle size distribution.

When selecting the filler, emphasis was placed on low values of the content of fine particles, the content of chloride salts and voids, and higher values of bulk density. From the considered spectrum of siliceous sands, sand of fraction 0–1 mm marked PR 31 (Provodín sands, Czech Republic) was chosen as the material with the highest preference, closely followed by sand of fraction 1–2 mm marked PR 1/2 (Provodín sands, Czech Republic). Fine quartz powder from the manufacturer Sklopísek Střeleč (Czech Republic) marked ST2 was chosen as a fine filler to complement the grain size curves. Composite gradation of fine aggregate from testing on Malvern Mastersizer is as follows (Figure 1).

To reduce the water coefficient and, thus, the mixing water used in the proposed mixture, and to increase the physical–mechanical properties, a superplasticiser based on ether polycarboxylate, labelled Peramin CONPAC 500 (IN-CHEMIE Technology, Olomouc, Czech Republic) was chosen in combination with an anti-foaming agent labelled AGITAN P 8850 (IN-CHEMIE Technology, Olomouc, Czech Republic). Both will be used in dry powder form and homogenised in a dry mixture.

As a polymer additive to optimise the utility properties of the final material, a styrene acrylate-based compound designated NEXIVA CT 714 (Redrock Construction s.r.o., Prague, Czech Republic) was used. A methylcellulose-based material labelled Tylose MH 6000 YP4 (Ceiba, s. r. o., Brandýs n. L., St. Boleslav, Czech Republic) was selected as an additive for rheology modification.

### 2.2. Casting Samples and Experiment Design

**Figure 2:**
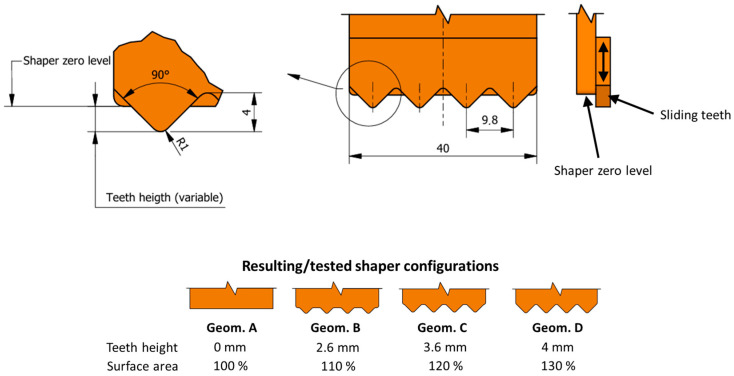
Teeth ejection mechanism and geometry of shaper.

The geometry of the interlayer surface area is formed by interlayer shapers. Shaper geometry was adapted from a study by He et al. [6], where the authors recommended a shaper with a tooth angle of 90° (or 45°) on a rectangular nozzle to increase interlayer strength. The authors considered a larger tooth angle of 45° more suitable than 30°, as it affects crack propagation across the specimen and, thus, achieves higher interlayer strength. Geometry with triangular teeth (height 4 mm, angle 90°, pitch 9.8 mm, see Figure 2) with curved vertices was chosen for this study. Various interlayer surface areas can be achieved by adjusting the depth of the teeth in the printed layer, simulating the process of the teeth ejecting and retracting. This was done to investigate the potential and significance of using such a device in future applications. The depth of the teeth in the printed layer was set to increase the surface area in 10% intervals. For instance, compared to a printed layer without teeth, slightly ejected teeth lead to an increase in surface area by 10%, 20%, and 30%.

As mentioned above, an experimental approach of casting samples was chosen. Thus, a form made of plywood was assembled, enabling the casting of specimens with different interlayer area geometries and time gaps between layers.

To ensure the majority hydration of the cement slurry products, the samples were stored for 28 days in water before mechanical properties testing.

### 2.3. Sample Preparation and Testing Conditions

**Figure 3:**
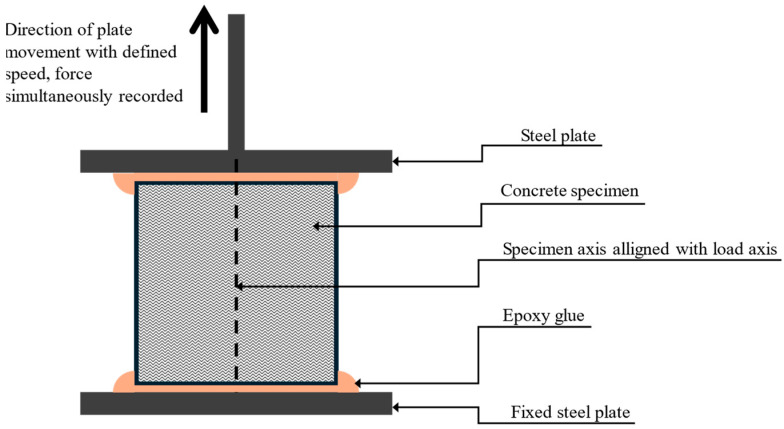
Uniaxial tensile test scheme.

From the cast samples, two types of test specimens were cut out (2 pieces of cubes 40 × 40 × 40 mm and 1 beam 40 × 40 × 160, width × height × length). The dimensions of the samples are not standardised; however, engineering stress can be easily calculated from the specimen’s geometry. The sides of each specimen were aligned by sawing under water, followed by 2 h of drying at 50 °C. After that, multi-material epoxy glue Sikadur 31+ was applied to connect the samples to steel profiles (anchors). After sufficient solidification of the epoxy glue, samples were tested in a uniaxial tension test conducted in a test press (designed according to ČSN EN 12390-4 [33]) with a loading speed of 0.5 mm/s (Figure 3). This value was chosen to ensure sufficiently slow loading, with reference to previous research, where loading speeds range from hundredths of mm/s to lower tens of mm/s [13,21,34], or approximately 0.035 MPa/s according to ASTM standards [27,35].

### 2.4. Data Evaluation

**Figure 4:**
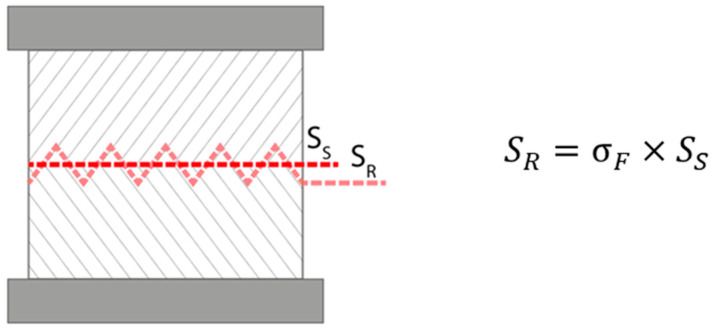
Graphical representation of real and simplified interlayer area.

During data evaluation, two approaches to calculating strength were used. Both calculations are based on the basic equation for engineering stress, σ=F/S, where σ is the stress, F is the applied force, and *S* is the cross-sectional area in the direction of applied force. The first approach uses a simplified interlayer area (S_S_ on following picture), not considering the teeth geometry. This method appears to better distinguish the strength of different geometries graphically and was, therefore, used in the following plots. The second approach uses the real interlayer area (S_R_), including the contributions of the teeth (see Figure 4). To mathematically describe the difference, the so-called shape factor (denoted σ_F_ (%)) can be used.

### 2.5. Microstructure Investigation

For a better understanding of the properties and sufficient description of mixture, the microstructure of the samples was examined using DTA (OZM Research s.r.o., Blížnovice, Czech Republic) and PANalytical Empyrean(Malvern Panalytical, Great Malvern, Worcestershire, UK) (Cu K-α radiation generated at 45 kV and 40 mA) and a scanning electron microscope TESCAN MIRA3 XMU (TESCAN GROUP, a.s., Brno, Czech Republic) operating at 30 kV. The samples for microstructure investigation were stored at a temperature of 23 ± 2 °C and a relative humidity of 55 ± 5%.

## 3. Results

**Figure 5:**
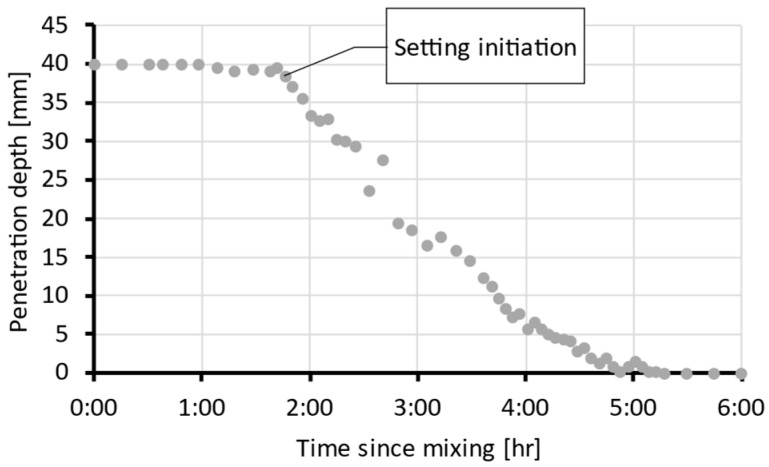
Indenter penetration depth over time from the Vicat test (representative curve from four replicates).

Before casting and testing, the mixture (Section 2.1) was subjected to the Vicat test to determine the initial and final setting times. Figure 5 shows the measured indenter penetration depth over time. The initial setting time was observed at approximately 1.75 h and the final setting time at 5.25 h. Based on these values, delay time intervals were selected for the first test series on an hourly scale.

### 3.1. Method Validation

**Figure 6:**
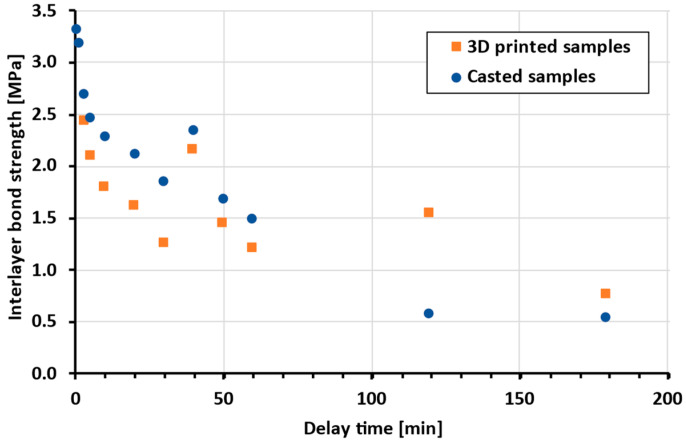
Comparison of bond strength of printed and cast samples (the same batch of material was used).

As mentioned, the experimental approach of casting samples was considered as more suitable. However, the authors wish to eliminate any doubts regarding the comparison of these methods; therefore, the bond strength of printed and cast samples with different delay times was tested (Figure 6).

The microstructure of selected cast and printed specimens was also examined, using differential thermal analysis (DTA), X-ray diffraction analysis (XRD), and a scanning electron microscope (SEM).

**Figure 7:**
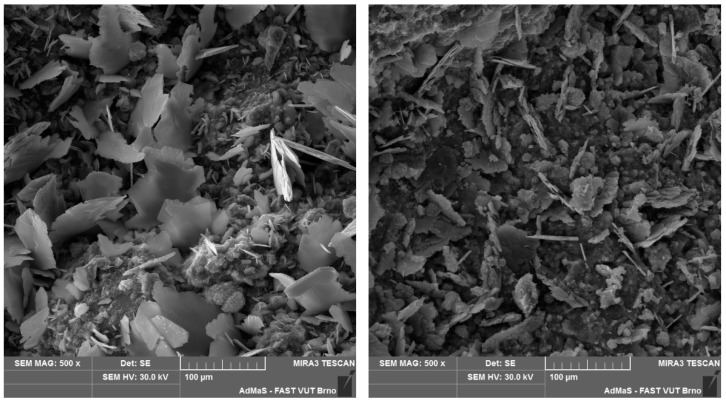
First (**left**) and second (**right**) layer of cast specimen with 120 min delay time under SEM.

**Figure 8:**
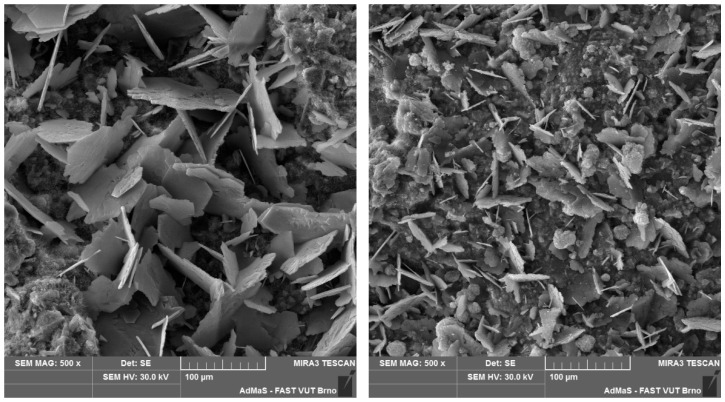
First (**left**) and second (**right**) layer of printed specimen with 120 min delay time under SEM.

According to the DTA results, all specimens contained comparable amounts of calcium silicate hydrate gel, portlandite and calcite. Furthermore, XRD analysis confirmed the identical mineralogical composition of all specimens. SEM analysis showed differences in the interlayer area in the size of CaCO_3_ crystals formed on the first and second layers, with the crystals formed on the first layer being larger. However, the distinction between cast and printed specimens was imperceptible (see Figure 7 and Figure 8).

### 3.2. General Delay Times

The first series of results was obtained with delay times selected based on the Vicat test. Delay times were chosen at 1-h intervals, to cover the entire setting time determined by the Vicat test. Twenty specimens were cast as described in Section 2.2, and each specimen was prepared according to Section 2.3.

### 3.3. Additive Manufacturing Delay Times

The second test was modified to prioritise practical conditions relevant from an AM perspective. The delay time between layers was reduced to values closer to those used in AM applications, where subsequent layers are typically deposited within minutes, rather than seconds.

**Figure 9:**
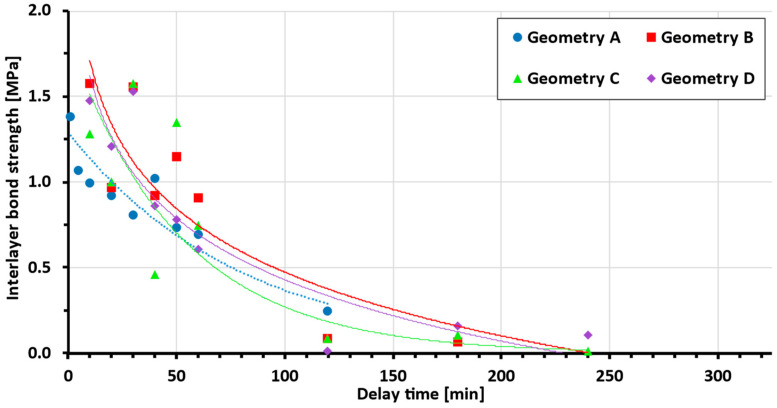
Bond strength vs. delay time, experimental data, and exponential trend line approximation (each geometry trend line is represented by a line of the same colour as the dataset).

After testing the interlayer bond of both sample groups, in accordance with Section 2.3, the following data shown in Figure 9 were obtained (for statistical details, see Table 2).

Figure 9 shows that the presence of teeth in different ejection states improves the bond strength between layers in both test setups. Geometries with teeth show an increase in bond strength of 30–45% (Geom. B, C, D) compared to interlayer geometry A (without teeth). Only samples that fractured between two layers were considered (samples in which adhesive–concrete interface failure occurred were discarded), and several samples were also damaged during preparation, resulting in some missing data points.

**Figure 10:**
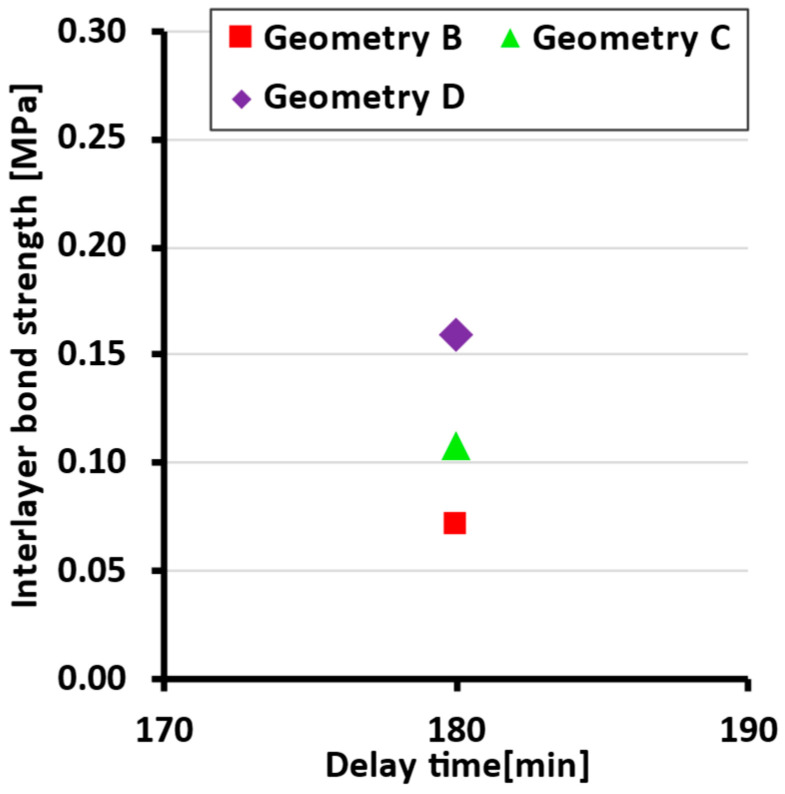
A zoomed-in section of the previous figure is shown (180 min delay time; geometry A is not plotted due to a zero value), with error bars replaced in Table 3 with minimum and maximum values.

When focusing on isolated data for specific delay times, it can be observed that the bond strength of the samples generally increases from the lowest interlayer surface area with the lowest bond strength to the highest bond strength for samples with a larger surface area. However, this expectation was not met in all cases (Figure 10), as will be explained later.

**Table 3:** Data overview for Figure 10.

Geometry	σ_F_ [%]	Min [MPa]	Displayed Value [MPa]	Max [MPa]
A	100	--	--	--
B	110	0.02	0.07	0.21
C	120	0.04	0.11	0.32
D	130	0.09	0.16	0.26

## 4. Discussion

Our comparison of the experimental approaches indicates that cast and printed samples are comparable in terms of interlayer characteristics, particularly bond strength and microstructure, within the investigated range of interlayer surface areas. This is also confirmed by the Student’s *t*-test, where for datasets of cast and printed bond strengths, the *p*-value is 0.27. This high *p*-value indicates that the tested groups of data (cast and printed) are comparable. Nevertheless, it is clear that the 3D-printed samples achieve lower bond strength. This may be caused by the oval geometry of the printed samples, where in the cross-section, the interlayer resembles a notch, which can promote crack initiation (as reported by He [6]). From the SEM results of both cast and printed samples, the only difference between the microstructure of the first and second layers after splitting (Figure 7 and Figure 8) is obvious. From a visual comparison of SEM images of the first layers, as well as XRD and DTA analyses, no statistically important difference can be found.

**Figure 11:**
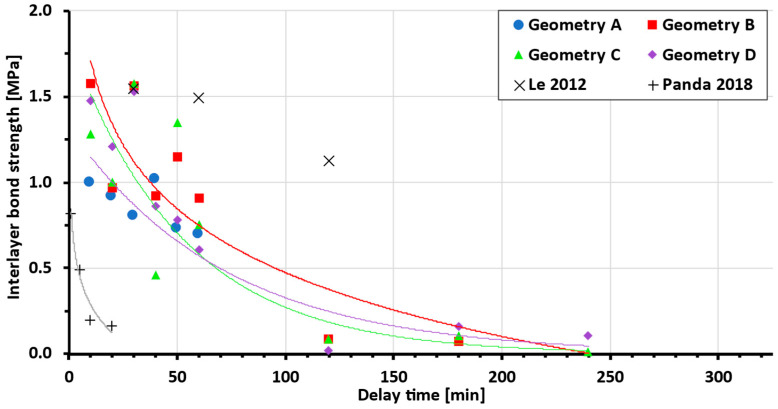
Comparing tested mixture with standard [27] and high-performance concrete [36] (each geometry trend line is represented by a line of the same colour as the dataset).

Data show a clear dependency of the interlayer bond strength on delay time across different interlayer geometries. From a comparison with other authors’ data (Figure 11), better performance of the tested (newly developed) mixture compared to the basic mixture [27] can be seen. Even though the bond strength increases at short delay times to the scale of high-performance concrete [36], the dependency on the delay time is much higher. Thus, further mixture optimisation can be achieved.

As shown in the results, the geometry with teeth shows an average increase of between 30 and 45% (geom. B, C, D) compared to interlayer geometry A (without teeth). This strength improvement corresponds well with He [6], who reported a 55% increase in bond strength with similar teeth geometry and side trowels. In the first test setup (delay time several hours), there was an obvious decrease in the layer bonding, which led to sample brittleness. Several times, the samples were damaged during sawing or did not even achieve manipulation strength. Therefore, not all geometries with delay times over 120 minutes were measured and are missing in Figure 9. This is one of the reasons why geometry *A* lacks measurement points over 1 h and why it was not possible to obtain a reliable approximation with a trend line. From the few successful measurements of geometry *A*, it is clear that the bond strength is lower compared to geometries with teeth. As mentioned in Section 3, only samples broken between two layers are shown.

**Figure 12:**
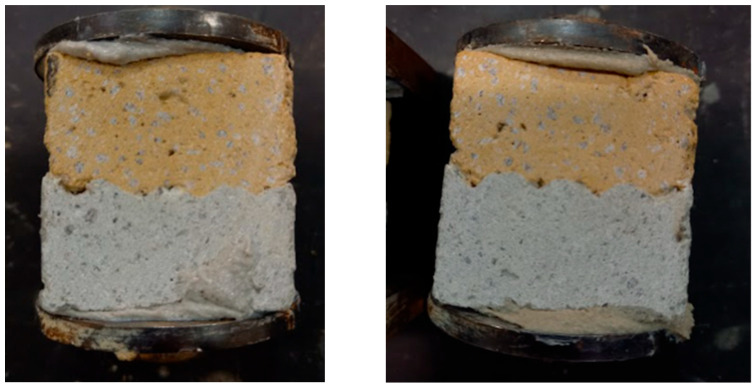
Cross-section of glued samples. Interlayer geometry B (**left**) and C (**right**). Yellow pigment added to the top layer for differentiation.

One discrepancy or irregularity can be observed in the plotted data. It was expected that more embossed teeth would lead to an increase in bond strength, and in each delay time, the strength would be ranked in the order D (strongest), C, B, A, as shown in Figure 10. In multiple cases, this order was not followed, and in the case of a delay time of 30 minutes, the points of geometries with teeth (B, C, D) almost merge at one point, beyond the resolution of measurement. This is probably caused by the imperfect tooth shape in the material (due to tearing or other errors arising during shaping). However, on most samples, a different tooth geometry between layers was visible on the cutting surface due to the use of 1.5% pigment (Figure 12), and with the exception of a 40-minute delay time, geometry A showed the weakest bond.

As mentioned above, the shaping of the interlayer geometry was not perfect. It should be noted that, as presented by Ji [20], the overall porosity and interlayer porosity can vary. It was observed that, while using the interlayer geometry with teeth, cracks in fresh concrete formed. Due to the plasticity of the concrete, the geometry was not smooth, and some material was torn out. Material tearing can cause higher porosity, and, therefore, the porosity and microstructure in the interlayer area need to be further investigated. If the tearing can be solved, for example, with different material or by modifying the shaper edges, an additional increase in the bond strength can be achieved. The mechanical performance of concrete is directly influenced by its porous structure [13,18]. As shown by Xiao [18], the volume fraction of pores with different sizes in the interlayer area can vary depending on the composition of the mixture, e.g., due to the incorporation of recycled fine aggregates or PE fibres. The structure and shape of the pores are also influenced by printing height, and, thus, further tuning of printing parameters and mixture with use of presented technology can lead to further increases in bond strength. It remains uncertain to what extent the observed improvement is solely the result of an increase in the contact area, or to what extent it is also influenced by changes in crack path deviation and stress redistribution. The use of Digital Image Correlation can be used in further research to deepen our understanding of crack propagation changes across different interlayer geometries (methodology, e.g., as presented by Xiao [37]).

The two approaches described in Section 2.4 were used to evaluate the data. The use of simplified interlayer area S_S_ leads to a better distinction of the strength of different geometries and, thus, was used in previous figures. While using real interlayer area S_R_, a more accurate representation of points in each delay time is achieved. This is consistent with the fact that each material has its own specific interlayer strength, due to which all geometries should converge at a single point for a given delay time. In no case was full convergence observed, which can be caused by affecting crack propagation across the section (further research required) or simply due to the limitations of experimental setup resolution.

**Figure 13:**
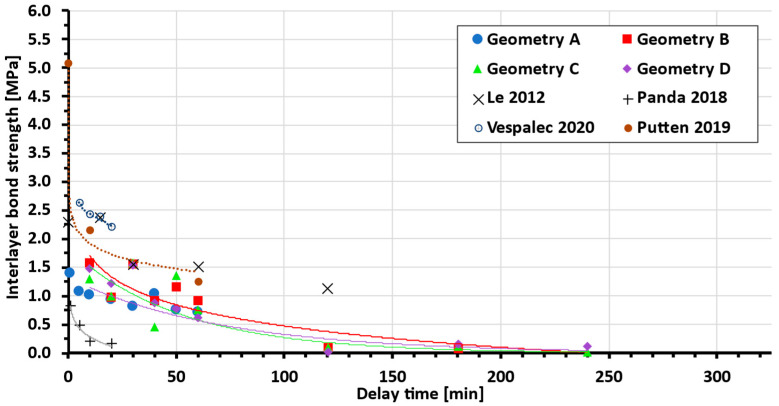
Comparison of measured data to other studies (for interpolation function, see Table 4; each geometry trend line is represented by a line of the same colour as the dataset) [13,27,36,38].

It is expected that for specific AM concrete material, master curve determining the delay time, interlayer surface area and the bond strength relationship will appear. In the current literature, the authors did not find agreement, whether the delay time and the bond strength relationship is determined by an exponential or logarithmic function (Figure 13). These functions, even though similar, can lead to very different proximity to data, and, thus, a large number of points in our and other authors’ studies is required.

As can be seen from Table 4 below, some authors reported only a limited number of bond strength measurements at different delay times, and, therefore, it is not clear which function is the most appropriate. Based on our data, the authors are leaning towards logarithmic dependence, due to more precise measurements and more data points in the section devoted to comparing printed and cast samples.

**Figure 14:**
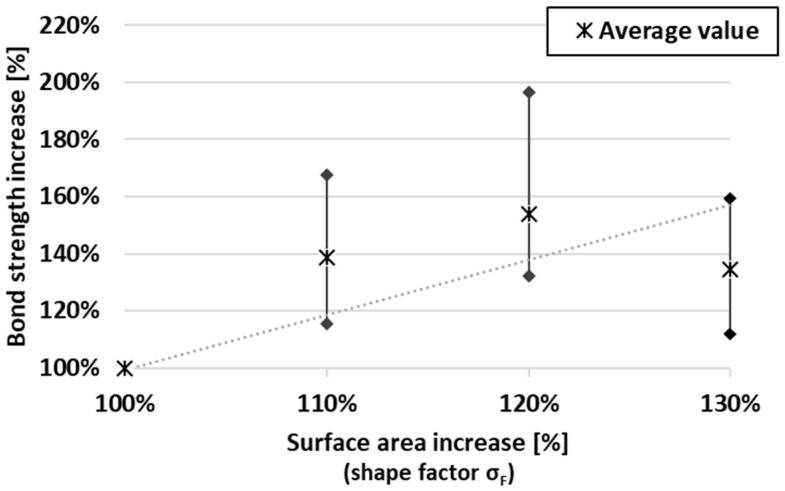
Plot of percentage increase in surface area and bond strength (the lower limit of the linear trend is represented by a dotted line).

Figure 14 shows the dependency of increasing interlayer surface area and an increase in bond strength (compared to geometry A). It needs to be acknowledged that, from the presented plot, the dependency function cannot be deduced. Based on the partial data, the authors are considering two dependency scenarios, which are not necessarily mutually exclusive. The first possibility is the linear dependency of bond strength increase on the surface area increase. A similar slope of linear trend lines was obvious for multiple delay times, with very little offset. From this, it is deduced that these curves are led by an equation that determines this relationship independently of the delay time (when comparing the percentage increase). The linear master curve is also in accordance with Zhao [28] (observing on a scale of roughness). However, a simple linear dependence is not entirely consistent with the presented data. The initial linear trend is followed by a decrease in bond strength. This led to the idea of an effective range of increasing the interlayer area. When increasing the contact area of the layers, imperfections occur during shaping (due to tearing of material, etc.), and after the threshold, bond strength is significantly compromised. This effective range is expected to be affected by tooth geometry and material. The limited effective range of varying the interlayer area is also in accordance with Sheng [29], whose results show a decrease in compressive and shear strength as the tooth angle increases (above threshold 22°). Although the data are from experiments with alkali-activated mortars, a similarity in the physical behaviour on a limited scale can be considered.

For a future study, the experimental setup (mould and shapers) requires modifications. Shortening the delay time between pouring and forming the interlayer geometry will hopefully lead to more accurate results, and the experimental setup will, thus, more closely resemble the conditions of 3D printing. To mathematically describe bond strength dependency on interlayer surface area, delay times should be scaled maximally in 30 min intervals for times above 1 h. For a more in-depth mathematical clarification of the findings, a study with a wider dataset is recommended.

The mathematical relationship between surface area and bond strength can be effectively demonstrated by incorporating the concept of tooth geometry setting. In practical scenarios, an object may be printed with varying time intervals between layers in different regions. The use of the presented ejecting teeth geometry can be used to achieve more homogenous mechanical properties across the cured object. The proposed technology requires verification under realistic print conditions. It is also expected that the influence of the presented teeth geometry can vary based on the loading conditions (shear, etc.), and further research should follow.

## 5. Conclusions

This study focused on clarifying the relationship between the interlayer surface area and the bond strength of cementitious material designed for 3D-printing technology. Research was conducted by testing cast samples, with different interlayer surface areas. The surface area was increased by forming a teeth geometry in samples, before casting the second layer. Various interlayer geometries were designed to imitate the ejection of teeth, thus indicating the possible use of this technology. Interlayer surface area appears to significantly affect the interlayer bond. With modified geometry, up to a 70% increase in strength can be achieved, which is consistent with previous publications. It is expected that this improvement will be greater in, e.g., shear load mode. From the research findings, the following conclusions can be stated.

- Interlayer bond strength increases with increasing interlayer surface area across different delay times between printed layers. Although interlayer bond strength decreases exponentially with increasing delay time, the strength increase resulting from a larger surface area appears to remain approximately constant in percentage terms. This finding suggests the possibility of defining an equation describing the relationship between surface area and bond strength.
- The relationship between the percentage increase in surface area and the percentage increase in bond strength appears to be linear until a certain threshold is reached. This threshold is expected to depend on the tooth geometry and material properties.
- It was shown that the technology of ejecting teeth to create different interlayer surface areas can be used to achieve more homogeneous mechanical properties in 3D-printed objects.

A mathematical description of this relationship and the application of the presented technology under various loading conditions will be the subject of further research. Such work may lead to more predictable printing results and support the wider adoption of 3D-printing technology with cementitious materials.

## 6. Patents

Practical application of modifying interlayer surface area is the subject of a utility model CZ 38510 U1 [40].

## Figures and Tables

**Table 1:** Mineralogical composition of used cement.

Mineral	Percentual Representation
C3S	55.3%
Calcite 1	19.7%
C4AF	7.8%
C2S	5.0%
Calcite	4.0%
Gypsum	3.0%
C3A cub	1.7%
Hemihydrate	1.5%
C3A ortho	1.0%
Arcanite	0.5%
Portlandite	0.3%
Quartz	0.3%

**Table 2:** Source data for Figure 9.

	Interlayer Bond Strength [MPa]											
	Geometry A			Geometry B			Geometry C			Geometry D		
Delay Time [min]	Min	Avg.	Max	Min	Avg.	Max	Min	Avg.	Max	Min	Avg.	Max
10	0.78	1.00	1.21	1.29	1.58	1.88	1.25	1.29	1.32	1.39	1.48	1.56
20	0.69	0.92	1.06	0.94	0.97	1.00	0.82	1.00	1.19	0.81	1.21	1.61
30	0.75	0.81	0.85	1.25	1.56	1.88	1.14	1.58	2.01	1.43	1.53	1.64
40	0.82	1.02	1.12	0.75	0.92	1.09	0.36	0.46	0.56	0.86	0.87	0.88
50	0.66	0.73	0.78	1.13	1.15	1.18	1.11	1.35	1.59	0.75	0.78	0.81
60	0.62	0.65	0.69	0.61	0.91	1.13	0.57	0.75	1.06	0.57	0.61	0.63
120	0.18	0.25	0.29	0.09	0.09	0.18	0.02	0.09	0.25	0	0.02	0.05
180				0	0.07	0.21	0	0.11	0.32	0.09	0.16	0.26
240							0	0.01	0.04	0.06	0.11	0.26

**Table 4:** Current studies: delay time and bond strength dependency function.

Author		Author-Determined Dependency Function (NA—Not Assigned, BF—Best Fit)	Number of Tested Delay Times	Note
Le 2012 [36]		exponential	11	High-performance printing concrete
Panda 2018 [27]		logarithmic	4	
Panda 2018 [39]		exponential	5	Geopolymer mortar
Vespalec 2020 [13]		NA, BF: logarithmic	4	
Putten 2019 [38]		NA, BF: logarithmic	3	
Presented study	Printed/cast samples	BF: logarithmic	12	See Figure 6
	Cast geometries A-B	BF: exponential	9	See Figure 9

## Data Availability

The original contributions presented in the study are included in the article, further inquiries can be directed to the corresponding author.
